# Impact of ZnS/Mn on the Structure, Optical, and Electric Properties of PVC Polymer

**DOI:** 10.3390/polym15092091

**Published:** 2023-04-27

**Authors:** A. M. El-Naggar, Zein K. Heiba, A. M. Kamal, Omar H. Abd-Elkader, Mohamed Bakr Mohamed

**Affiliations:** 1 Research Chair of Exploitation of Renewable Energy Applications in Saudi Arabia, Physics & Astronomy Department, College of Science, King Saud University, P.O. Box 2455, Riyadh 11451, Saudi Arabia; elnaggar@ksu.edu.sa; 2 Physics Department, Faculty of Science, Ain Shams University, Cairo 11566, Egypt; 3 Physics & Astronomy Department, College of Science, King Saud University, P.O. Box 2455, Riyadh 11451, Saudi Arabia

**Keywords:** PVC, ZnS/Mn, preparation temperature, structure, optical, electric, activation energy

## Abstract

The most efficient way to create novel materials that may be used in a variety of optoelectronic applications is thought to be doped mixed polymers with appropriate fillers. Undoped and doped PVC polymers with ZnS/Mn formed at different temperatures were fabricated using the casting method. The Rietveld method was used to discover the structure and microstructure of Zn_0.95_Mn_0.05_S prepared at T = 300, 400, and 500 °C. The distribution and existence of the nanofiller over the PVC matrix were determined via XRD, FTIR, EDS, and SEM techniques. The effect of the preparation temperatures of the ZnS/Mn nanofiller on the absorption, transmittance, reflectance, refractive index, extinction coefficient, dielectric constant, AC conductivity, electrical modulus, and DC conductivity activation energy data of the PVC polymer was studied using the diffused reflectance technique. Doping PVC with ZnS/Mn (prepared at 300 °C) lowered the direct and indirect optical band gaps from 5.4 and 4.52 eV to minimum values of 4.55 and 3.63 eV. The fluorescence intensity of pure PVC is greatly enhanced upon loading with ZnS/Mn. The PVC exhibited two near UV peaks, one violet and one blue color, while, in addition, the doped polymers exhibited green and orange colors. The corresponding CIE diagram for all the samples was also determined.

## 1. Introduction

Organic–inorganic hybrid optical materials are extremely valuable from a technological perspective. Hybrid compositions with remarkable properties may emerge once the relationships between the properties of inorganic elements and polymer matrices are clarified. Polymeric nanocomposites, because of their interesting properties, are also a significant class in the field of applied materials science technology. Polymer composites display a variety of fascinating optical features, including a high/low refractive index, tailored absorption/emission spectra, and strong optical nonlinearities. Hybrids are eligible for potential optoelectronic applications because they possess such rare properties [1,2,3,4].

Polyvinyl chloride (PVC) is a popular thermoplastic polymer because of its many useful properties, including its low flammability, chemical resistance, and inexpensiveness [5].

The binary semiconductor compound zinc sulfide (ZnS) has a large band gap energy and high transmission. Depending on the conditions under which it is deposited, ZnS can conduct electricity as either n- or p-type. Direct optical band transitions can be seen in both the zinc blende and hexagonal wurtzite crystal structures, which are the most common for ZnS growth. ZnS is a semiconductor with a large band gap, measuring 3.77 eV for hexagonal structures and 3.72 eV for cubic ones. The use of ZnS with either n- or p-type electrical conductivity is crucial in the manufacturing of electronic devices such as solar cells [6]. White light-emitting diodes (WLEDs) made with the obtained Mn-doped ZnS/ZnO heterostructures show great promise [7]. Photovoltaic solar cells and optoelectronic devices are two potential fields where ZnS:Mn films could prove useful [6]. The optical band gap of ZnS exhibited a blue shift as it was doped with Mn [8].

For the purpose of protecting against X-ray radiation, Gholamzadeh et al. prepared barium-doped PVC/Bi_2_WO_6_ composites [9]. Maksoud et al. irradiated Cu/Cu_2_O nanorods/PVC with gamma irradiation to be used in energy storage applications [10]. Tin-doped ZnS@ polyvinyl pyrrolidine polymeric composite films with altered photoluminescent and electrical properties were developed by Badawi et al. for use in light-emitting diodes [11]. The preservation of the optical properties of the incorporated nanoparticles makes these nanocomposites promising candidates for optoelectronic devices, and the thermomechanical stability of the Cr-doped ZnO-based PVC nanocomposite samples may alter the traditional roles of PVC polymers from insulators to those of materials for energy storage and capacitors [12]. PVC-doped iodine thin films’ optical, chemical, and thermal properties can be modified by incorporating an appropriate concentration of iodine nanofiller into the PVC polymeric matrix [13]. Synthesized modifications to zinc oxide nanoparticles with polyethylene glycol (6 kDa) improved the performance of PVC membranes. PVC membrane morphology and hydrophilicity were modified by nanoparticle zinc oxide incorporation. The addition of zinc oxide nanoparticles to modified PVC membranes increased their water flux and rejection of IBP [14]. One-step electrospinning was used to successfully prepare PVC nanocomposite films containing ZnO nanoparticles, and corrosion electrochemical tests showed that the nanocomposite PVC–ZnO structures could be heated to temperatures 20 °C above the glass transition temperature of the polymer to achieve superior anti-corrosion behavior [15]. Photocatalytic research demonstrates that PVC/CdS nanocomposite films have promising applications in the degradation of MB when exposed to UV light [16]. According to research conducted by Rasmagin et al., who investigated the optical characteristics of nano CdSe/ZnS in heat-treated PVC films, heating for a short period of time increases the photoluminescence intensity, while heating for a long period of time significantly decreases it [17]. Furthermore, Thai et al. discovered that polyvinyl alcohol-coated ZnS:Mn nanoparticles increased both the photoluminescence and the crystal field of Mn^2+^ ions [18]. The nonlinear optical (NLO) features were improved with an increase in the Mn:ZnS doping percentage in Mn: ZnS/PMMA polymer nanocomposites [19]. The high values of the NLO parameters in the upper part of the visible range, especially a PVA/CMC blend loaded with 0.5 MnS–0.5 ZnS, may nominate them for use in some fields, including fast optical switching, optical limiting, and high-speed communication [20]. Huong et al. discovered that electrospun polymer (PMMA or PAN) hybrids with Mn-doped ZnSe/ZnS nanocrystals had enhanced luminescence [21].

Previously, we studied the influence of doping and variation in the annealing temperature on the structural and optical features of ZnS. It was found that the optical band gap of nano ZnS/Co or ZnS/Mn was first increased then decreased as the annealing temperature increased while in the case of ZnS/Fe, the optical band gap increased as the annealing temperature increased. Furthermore, the emission spectra were affected by the kind of dopant and the annealing temperature of the host matrix [22]. Furthermore, PVA/CMC/PVP polymer blends loaded with ZnS/Co (400 and 500 °C) showed the highest values for the refractive index. Both the calcination temperature of the nanofiller and the excitation wavelength have an impact on the emitted colors and intensities. Except for blends doped with ZnS/Co (300 °C), the real part of the dielectric constant decreased as the temperature rose. Doping the blend with ZnS/Co prepared at 400 and 500 °C led to an enhancement in AC conductivity, while loading it with ZnS/Co prepared at 300 °C resulted in only a minor shift in conductivity. ZnS/Co prepared at 400 and 500 °C raised the blend’s electric modulus, while ZnS/Co prepared at 300 °C reduced it [23]. In addition, a PVA/CMC blend loaded with ZnS/Fe formed at 300 and 400 °C showed promise as UV-blocking and UV-filtering materials. Doped blends’ optical band gaps are sensitive to the nanofiller formation temperature. As the blend was fortified with nanofiller made at 300 °C, the refractive index and nonlinear optical parameters rose to their highest values. Blends loaded with ZnS/Fe formed at 300 and 400 °C showed an increase in fluorescence intensity, suggesting their use as a promising material for LEDs and solar cells [24].

Therefore, the variation in the nanofiller formation temperature can alter its properties and, hence, change the properties of the host polymers. Furthermore, Mn ion has several oxidation states that can affect the temperature of preparation and, hence, affect the properties of ZnS. In order to create PVC/ZnS/Mn nanocomposite films, ZnS/Mn prepared at various temperatures were dispersed in PVC using the solution casting scheme. The structural, optical, and AC electrical characteristics of the PVC/ZnS/Mn nanocomposite films were investigated.

## 2. Methods and Materials

### 2.1. Chemicals Used

Zinc acetate dihydrate (Zn(CH_3_COO)_2_ · 2 H_2_O, Sigma Aldrich, St. Louis, MO, USA, 98%), manganese acetate ((CH_3_CO_2_)_2_Mn, Sigma Aldrich, 98%), thiourea (NH_2_CSNH_2_, Sigma Aldrich, 99%), and polyvinyl chloride ((CH_2_CHCl)_n_, Sigma-Aldrich, 99%).

### 2.2. Synthesis Procedure

To create the Zn_0.95_Mn_0.05_S (ZnS/Mn) sample, a stoichiometric mixture of zinc acetate, manganese acetate, and thiourea (0.95:0.05:1) was mixed and ground for 3 h. The powder was separated into three portions, each of which was heated in an electric oven for three hours at temperatures of 300, 400, and 500 °C (3 h). Below 300 °C, the ZnS/Mn sample did not form while beyond 500 °C, the ZnS/Mn sample is oxidized.

PVC polymer was created using the casting method, which involved dissolving 2 g of polyvinyl chloride in 40 mL of tetrahydrofuran (THF) at room temperature for 4 h until a clear solution was produced. Using the previous step, PVC/ZnS/Mn polymers were also created, but these were made with ZnS/Mn (5 wt%) that had been prepared at various temperatures. The ratios between the nanofiller to PVC were calculated according to the following relation:

$$x\;\left(\mathrm{wt}\%\right)=\frac{w_{f}}{w_{p}+w_{f}}\times 100$$

where *w_f_* and *w_p_* are the weights of the nanofillers and polymer, respectively.

**Figure 1:**
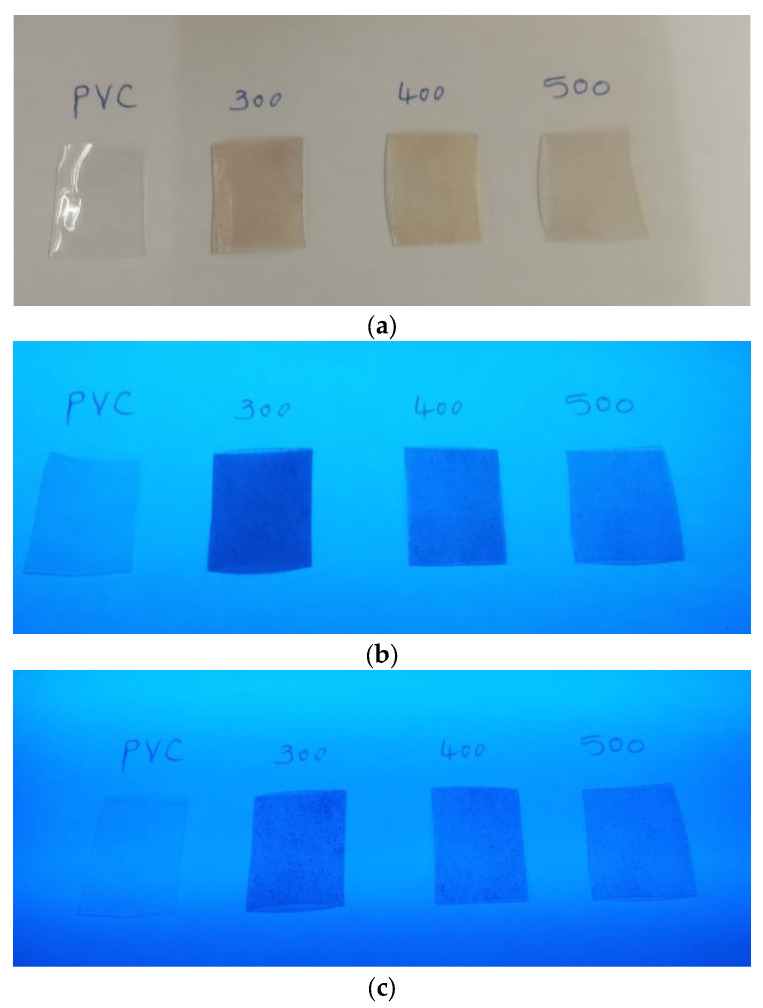
The photographs of all films under (**a**) normal light and UV light, (**b**) 254 nm, and (**c**) 365 nm.

The films were produced after the resultant solutions were placed separately in Petri dishes for two days, as shown in Figure 1. The formed polymers were 0.21 to 0.3 mm thick (performed using the digital micrometer).

The X-ray diffraction data were gathered using a PANalytical diffractometer (X’pert MPD, Philips, Cu source). The fluorescence spectra were acquired using a luminescence spectrophotometer (FP-8200 JASCO). Scanning electron microscope (SEM) images and EDS analysis were obtained using a JEOL (Akishima, Tokyo, Japan JED-2200 Series) instrument. Different vibrational bands in the system were identified using FTIR spectroscopy (Bruker Tensor 27 FTIR Spectrometer).

Diffuse reflectance spectrophotometer measurements (JASCO-V-670) with an attached integrating sphere assembly were used to obtain UV diffuse reflectance (*R*), absorbance (*A*), and transmittance (*T*) spectra for all samples. Before performing additional calculations, the *R* values were adjusted using the same way described in Ref. [25].

Using Tauc methodology, the direct and indirect optical energy gaps (*E_g_*) were computed [26]:

(1) $$\alpha h\upsilon =D{\left(h\upsilon -E_{g}\right)}^{q}$$

where *h*, υ, *D*, *d*, and α (=*A*/*d*) are Planck’s constant, the frequency of the incident light, a constant known as the disorder parameter, the thickness of the blended polymer absorption coefficient, respectively. *q* could be 0.5 or 2 for direct or indirect transition, respectively.

Using the relations in [27], the refractive index (*n*) and extinction coefficient (*k*) were calculated.

The dielectric constant in the frequency range (100 Hz–1 MHz) at room temperature for a polymer disk with diameter (*D*) = 13 mm and thickness (*t*) = 0.21–0.3 mm was determined by measuring the capacitance (*C*) and dissipation factor (tan δ) in response to applied AC voltages (2 V) using the LCR-8105G device.

Following the next formula [28], the real (*ε′*) and imaginary (*ε″*) dielectric constant parts and AC conductivity (*σ_ac_*) of various polymers were estimated:

(2) $$\varepsilon^{\prime }=dC/\varepsilon_{0}A$$

(3) $$\sigma_{ac}=2\pi f\varepsilon_{0}\varepsilon^{\prime }\tan\delta$$

(4) $$\tan\delta =\varepsilon^{\prime \prime }/\varepsilon^{\prime }$$

where *ε*_0_ is the permittivity of free space.

The electrical conductivity ($\sigma_{dc}$) is provided by the subsequent equation:

(5) $$\sigma =\frac{L}{R\cdot A}\;$$

where *L*, *A*, *R* ($=\frac{V}{I}$), *ε*_0_, *V*, and *I* are sample thickness (mm), electrode area (m^2^), resistance (Ω), permittivity of free space, voltage (volts), and current (amperes), respectively.

## 3. Results and Discussion

### 3.1. Structural Investigation

**Figure 2:**
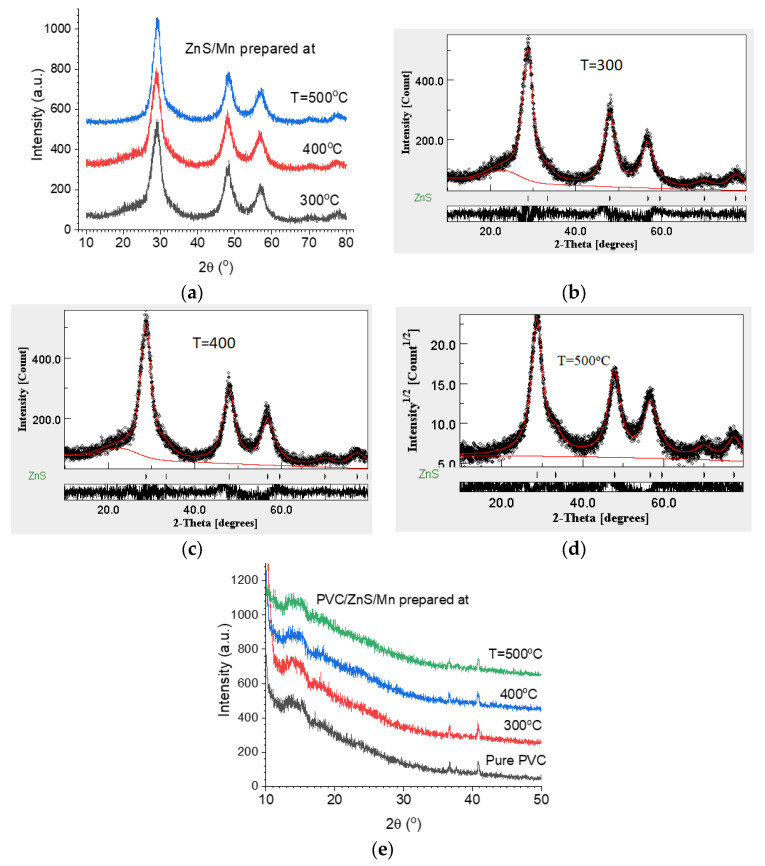
XRD diffraction patterns for (**a**) ZnS/Mn prepared at different temperatures, (**b**–**d**) Rietveld refinement of ZnS/Mn prepared at 500 °C, and (**e**) pure and doped PVC polymers with ZnS/Mn prepared at different temperatures.

The obtained diffraction patterns for the powder nanofiller Zn_0.95_Mn_0.05_S (ZnS/Mn) prepared at T = 300, 400, and 500 °C are illustrated in Figure 2a. All the samples are single phase ZnS with a zinc blende structure, space group $F\;\bar{3}4m$. The diffraction patterns were analyzed by applying the Rietveld method (Figure 2b–d) and the resulting refined parameters are given in Table 1. It can be seen that the crystallite size is increased a little upon raising the preparation temperature while the lattice microstrain is greatly reduced. The lattice parameter (*a*) is increased a little due to an increase in the crystallite size. Figure 2e depicts the diffraction patterns measured for the pure PVC and the PVC loaded with ZnS/Mn. All diffraction patterns have the same features as that of the pure PVC with no peaks characteristic of ZnS/Mn due to a small amount of ZnS/Mn. Owing to the poor crystallization of the PVC [29], the diffraction patterns exhibited a high background due to diffuse scattering being overlapped with two weak peaks at 36.7 and 41.2°, and a broad hump around 17°.

**Figure 3:**
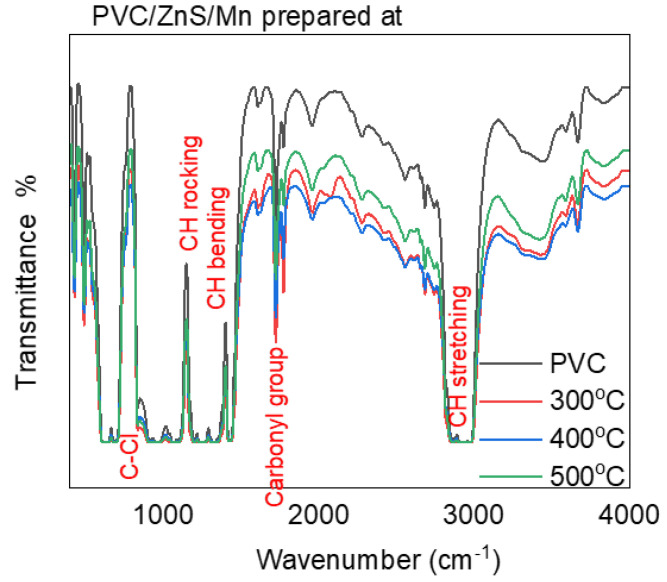
FTIR patterns for pure and doped PVC polymers with ZnS/Mn prepared at different temperatures.

Figure 3 displays the Fourier transform infrared (FTIR) transmittance data for the pure and ZnS/Mn-doped PVC polymers. The PVC polymer displayed C–Cl bond stretching, and carbonyl group vibrations at 790 and 1737 cm^−1^, respectively [30,31,32]. The PVC polymer’s CH stretching, CH bending, and CH rocking vibrations are located at 2912 cm^−1^, 1429 cm^−1^, and 1130 cm^−1^, respectively [33,34]. Upon doping the PVC with ZnS/Mn prepared at different temperatures, the peak positions and intensities of the vibrations changed slightly. This indicated the occurrence of an interaction between the PVC and doping substances.

**Figure 4:**
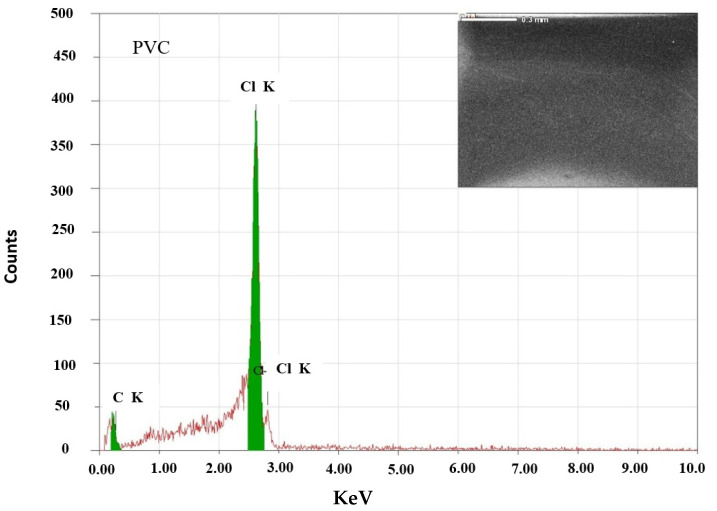

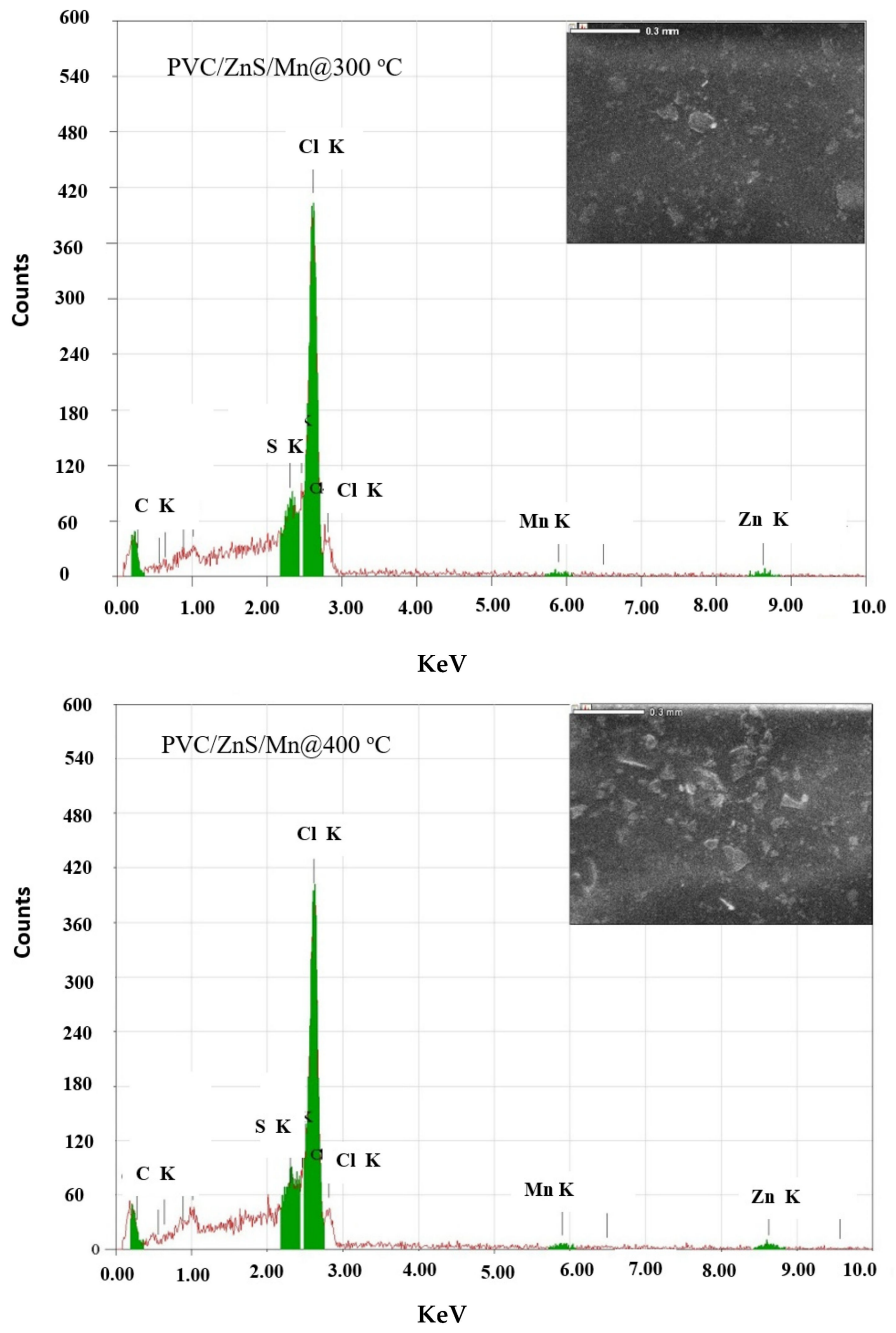

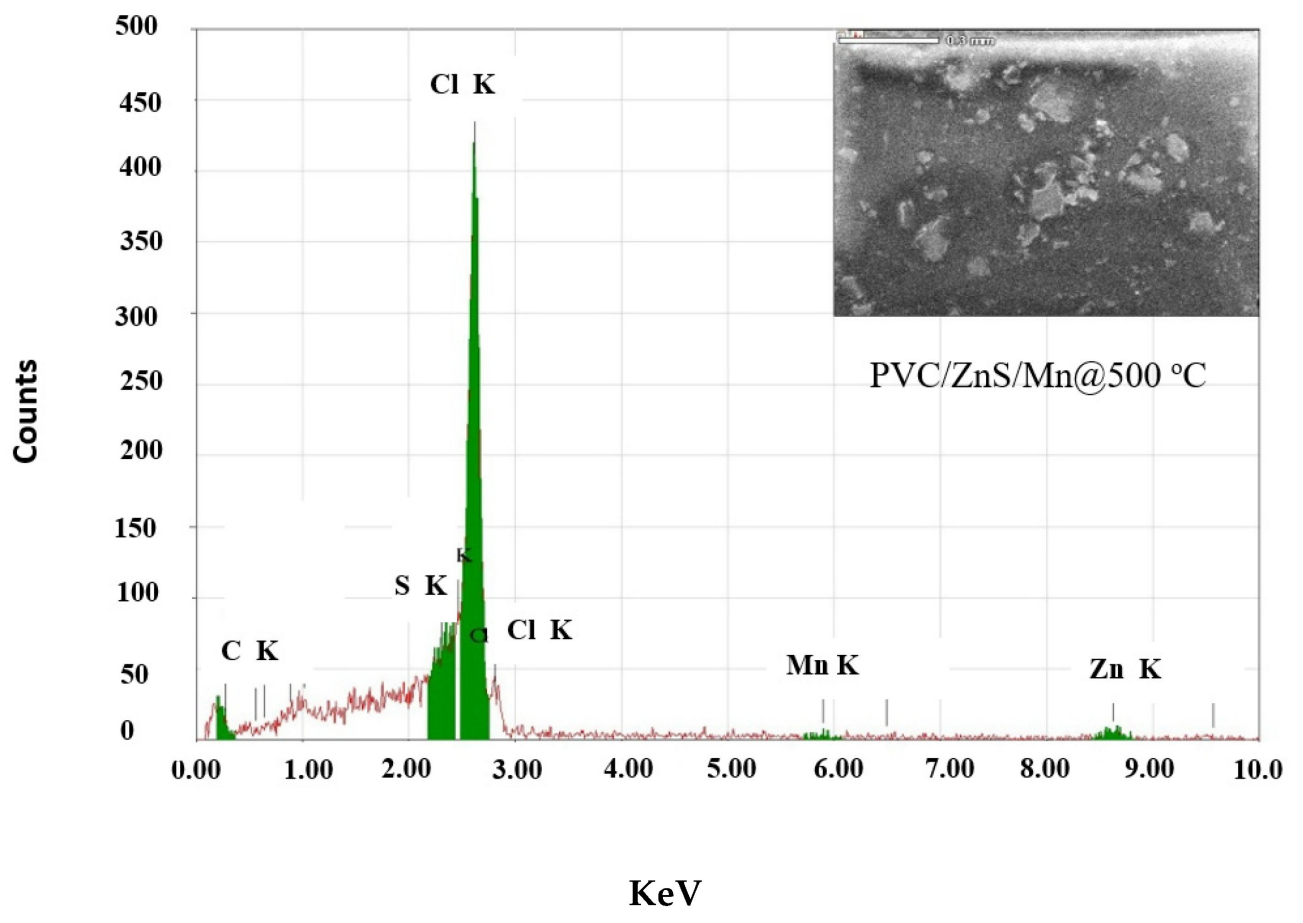
EDS and SEM patterns for pure and doped PVC polymers with ZnS/Mn prepared at different temperatures.

Figure 4 depicts the SEM images and EDS analyses of the PVC and PVC/ZnS/Mn polymers. The SEM micrograph of the pure PVC lacks pores and is smooth. The film surface morphology changed from smooth to rough when ZnS/Mn prepared at different temperatures was added to the PVC polymer, and the degree of roughness increased with the increasing ZnS/Mn preparation temperature. The EDS data of the PVC and PVC/ ZnS/Mn polymers prepared at different temperatures revealed the presence of (C, N, Cl) and (C, N, Cl, Zn, Mn, S) ions, respectively.

### 3.2. Optical Features

**Figure 5:**
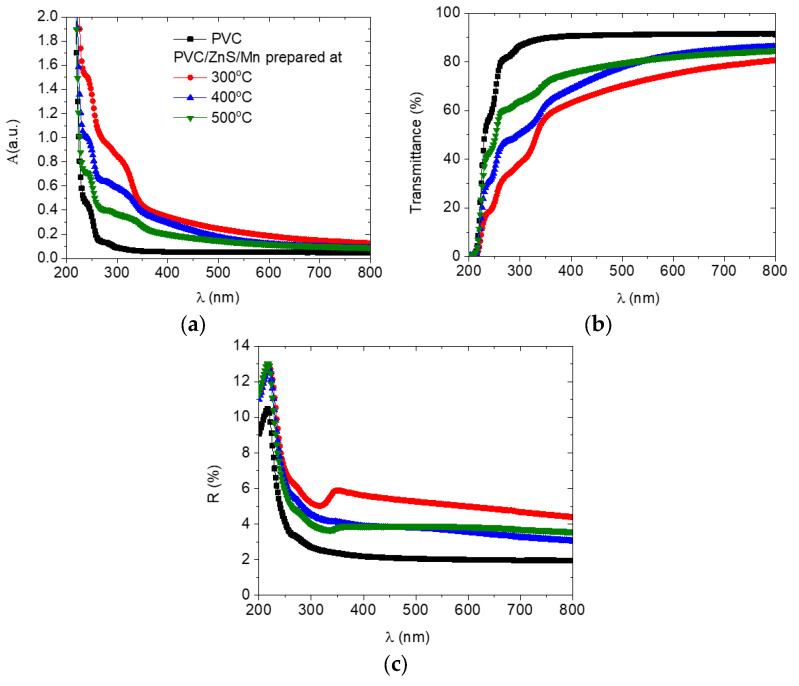
(**a**) Absorbance, (**b**) transmittance, and (**c**) reflectance spectra for pure and doped PVC polymers with ZnS/Mn prepared at different temperatures.

Figure 5 illustrates how a change in the preparation temperatures of the ZnS/Mn nanofiller affects the absorption, transmittance, and reflectance data of the PVC polymer. The polymer’s absorbance was improved in the 𝜆 range ≥ 240 nm upon loading with ZnS/Mn, as shown in Figure 5a. Due to the increase in absorbance upon doping, the doped polymers can be used as UV-blocking materials. In addition, the absorption band of the doped polymer is redshifted relative to the undoped polymer, and this shift increased as the nanofiller preparation temperature is reduced, as shown in Figure 5a. The PVC loaded with Cr-doped ZnO exhibited a similar feature [12]. In the doped polymers, a new molecular bond between cations and anions may be responsible for this change. As a result, defects form within the doped PVC matrix. As a result of the doping, the optical transmittance spectra of the doped polymers were reduced compared to that of the undoped PVC. The transmittance decreased significantly as the temperature of the ZnS/Mn synthesis was reduced, as shown in Figure 5b. The lowering in the transmittance spectra of the host polymer with a decrease in the temperature of the preparation of ZnS/Mn reflects the role of the particle size in the scattering of the incident beam either by volume or surface scattering [35]. Doping the PVC with ZnS/Mn improved its optical reflectance spectra, and the optimum value was reached when the nanofiller was prepared at 300 °C, as shown in Figure 5c.

**Figure 6:**
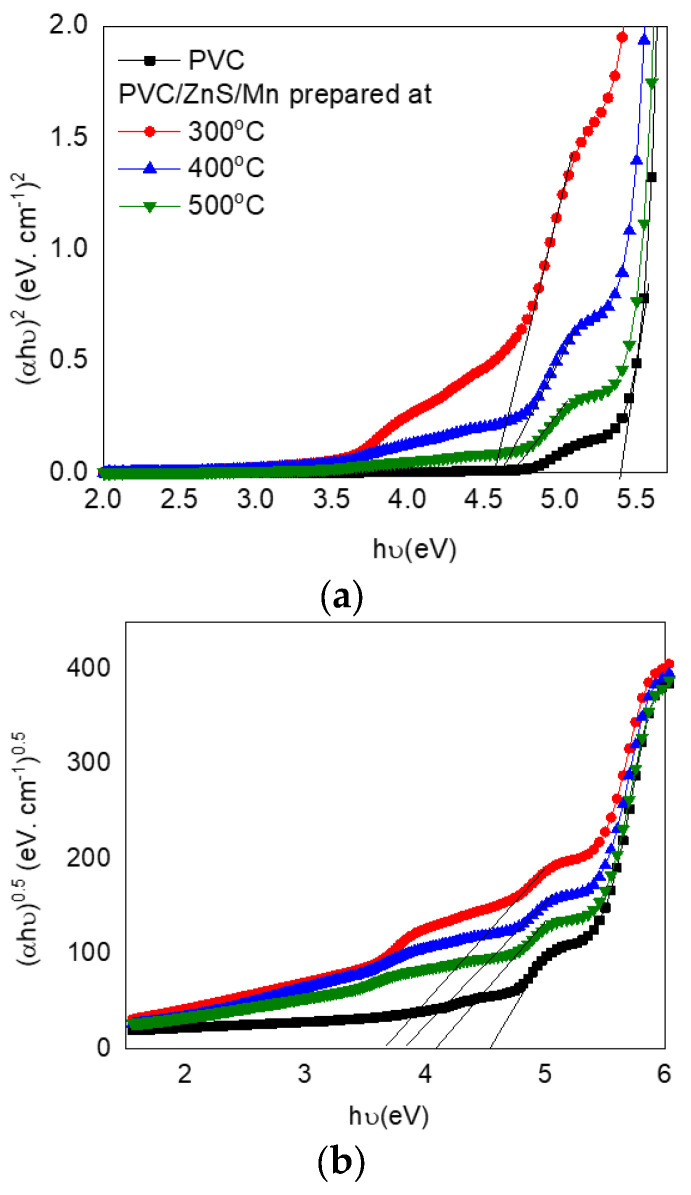
Tauc relation for (**a**) direct and (**b**) indirect optical band gaps for pure and doped PVC polymers with ZnS/Mn prepared at different temperatures.

For the permitted electronic transitions, the optical band gap energy (*E_opt_*) is as follows: The linear portion of Figure 6, which intercepts with the x-axis, is extrapolated to obtain the direct (*E_dir_*) and indirect (*E_ind_*) band gap energies. *E_dir_* and *E_ind_* were decreased from 5.4 and 4.52 eV to minimum values of 4.55 and 3.63 eV as the PVC was doped with ZnS/Mn (formed at 300 °C), as shown in Table 2. The *E_dir_* and *E_in_* values increased with an increase in the nanofiller’s (ZnS/Mn) preparation temperature. Upon altering the nanofiller’s annealing temperature, the degree of disorder in the blend and the number of nanofiller-induced defects within the host blend varied. Therefore, the optical band gaps were modified as a result of the introduced localized state.

**Figure 7:**
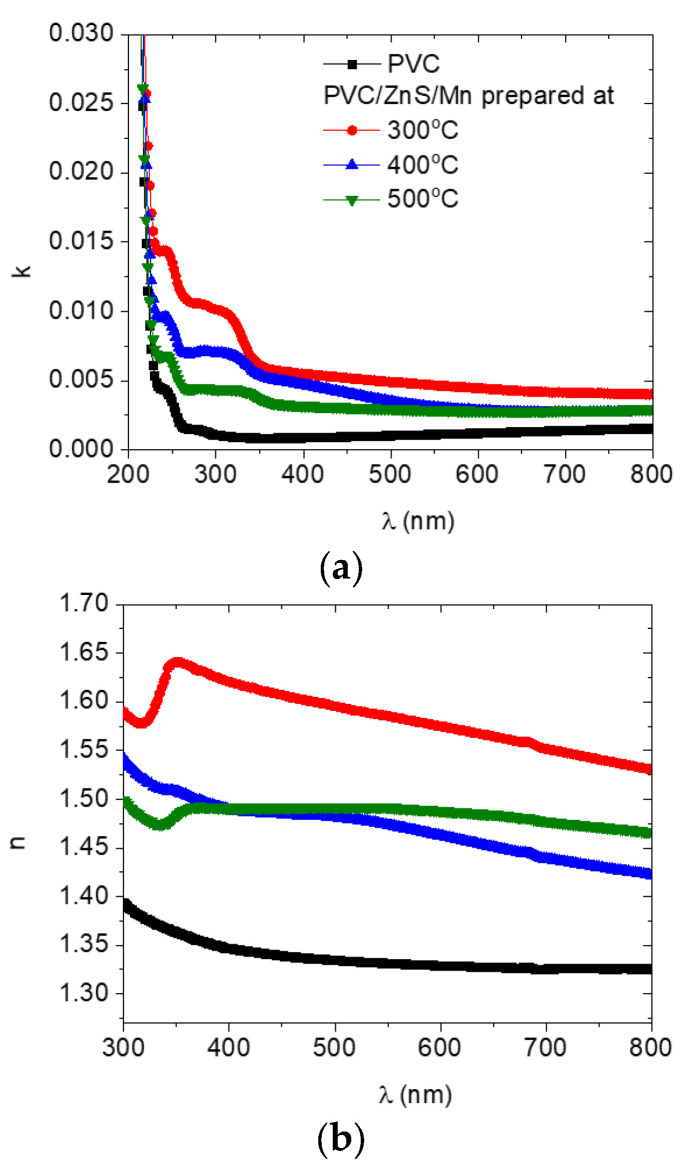
Variation in (**a**) extinction coefficient and (**b**) refractive index with the wavelength for pure and doped PVC polymers with ZnS/Mn prepared at different temperatures.

The extinction coefficient (*k*) and refractive index (*n*) values for all the polymers as a function of the wavelength are displayed in Figure 7. As can be seen in Figure 7a, as the wavelength increases, the values of *k* for all the polymers is reduced. As the PVC polymer was doped with ZnS/Mn, its *k* value increased. When ZnS/Mn was prepared at a temperature of 300 °C, the *k* value reached its maximum value and decreased as the preparation temperature rose. This change in the *k* value may be related to the polymer’s absorption behavior after doping with nanocrystal fillers, which changes the interaction between the incident light and free carriers in the doped blends [36].

The *n* value of the undoped and doped PVC polymers with ZnS/Mn formed at different temperatures is revealed in Figure 7b. The *n* data from the undoped and doped PVC with ZnS/Mn prepared at 400 °C showed a normal dispersion that decreased as the wavelength increased. Normal and abnormal dispersion behaviors were observed in the *n* value of the polymer doped with ZnS/Mn prepared at 300 and 500 °C. As the wavelength increased from 320 to 350 nm, it dropped, increased, and then dropped once more. The doped polymer has a higher *n* value than its undoped counterpart. Doping the PVC polymer with a nanofiller may alter its *n* value because of changes in the density of chain packing [37], as a result of changes in the intra–intermolecular bonding formed between the nano ZnS/Mn and the host polymer. The *n* value displayed the uppermost value in the PVC doped with ZnS/Mn formed at 300 °C. Changes in the number of oscillating dipoles are responsible for the redistribution of electronic charges and, consequently, the modification in the polarizability of the blends, which accounts for the variation in the *n* values of the various polymers [38].

### 3.3. Fluorescence Analysis

**Figure 8:**
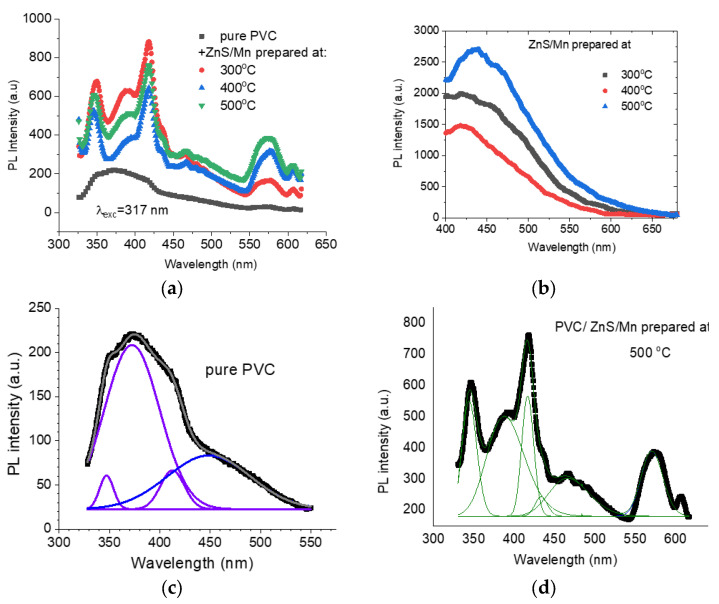
(**a**) FL spectra for pure and doped PVC polymers with ZnS/Mn prepared at different temperatures, (**b**) FL spectra for the nanofillers, and (**c**,**d**) Gaussian fitting for FL spectra.

The measured FL spectra of the PVC loaded with ZnS/Mn prepared at *T* = 300, 400, and 500 °C, under an excitation wavelength of *λ* = 317 nm, are depicted in Figure 8a. The FL intensity of the pure PVC is greatly enhanced upon loading with ZnS/Mn and well-resolved emission peaks appeared. For comparison, the FL spectra for the powder filler ZnS/Mn are represented in Figure 8b. As revealed from the graph, the FL intensity increased as the preparation temperature increased then decreased with the increase in the formation temperature of the nanofiller. The spectrum of the pure PVC, as shown in Figure 8c, manifested a broad fluorescence emission extended from 300 to 320 nm and comprises a broad peak with shoulders to the right. Decomposition of the spectrum yields two near UV peaks (347, 372 nm), one violet (412 nm), and one blue (448 nm). These emission peaks are resolved and become sharp upon loading ZnS/Mn, as shown in Figure 8d, for the decomposed spectrum of the PVC/ZnS/Mn prepared at 500 °C. The emission spectrum of the PVC is dominated by excimer fluorescence [39]. El-Hachemi et al. assigned the emission band, under an excitation of 467 nm, observed for pristine PVC at 467 nm to PVC excimer fluorescence corresponding to π*–π transition. Under 340 nm excitation, a wide hump-like emission band with a tip around 402 nm (violet) was observed for the pure PVA. For the PVC film, the FL emission spectrum under *λ*_exc_ = 310 nm disclosed a peak at 380 nm which is shifted to ~425 nm under an excitation of 360 nm [40]. Accordingly, the main emission band at 372 nm may be assigned to PVC excimer fluorescence corresponding to π*–π transitions [41]. The shoulder bands at 347 and 412 nm, which became pronounced for PVC/(ZnS/Mn), are most probably due to trapped energy levels created by impurities in the PVC [42].

The enhancement in the FL intensity, upon loading with ZnS/Mn, may be attributed to the presence of intermolecular interactions between the PVC molecules and ZnS. Sliva et al. [43] attributed the changes in the C–Cl stretching FTIR peaks of the PVC/ZnS composite to the interaction between the PVC chlorine atom and ZnS, which is confirmed by the PCA (principal component analysis) of the FTIR pure PVC and PVC/ZnS spectra. A similar enhancement was observed for a PVA/ZnS composite [44], and for PVP-coated ZnS [45]. Such an enhancement nominates the present system as having potential to be used for light-emitting diode and solar cell applications.

**Figure 9:**
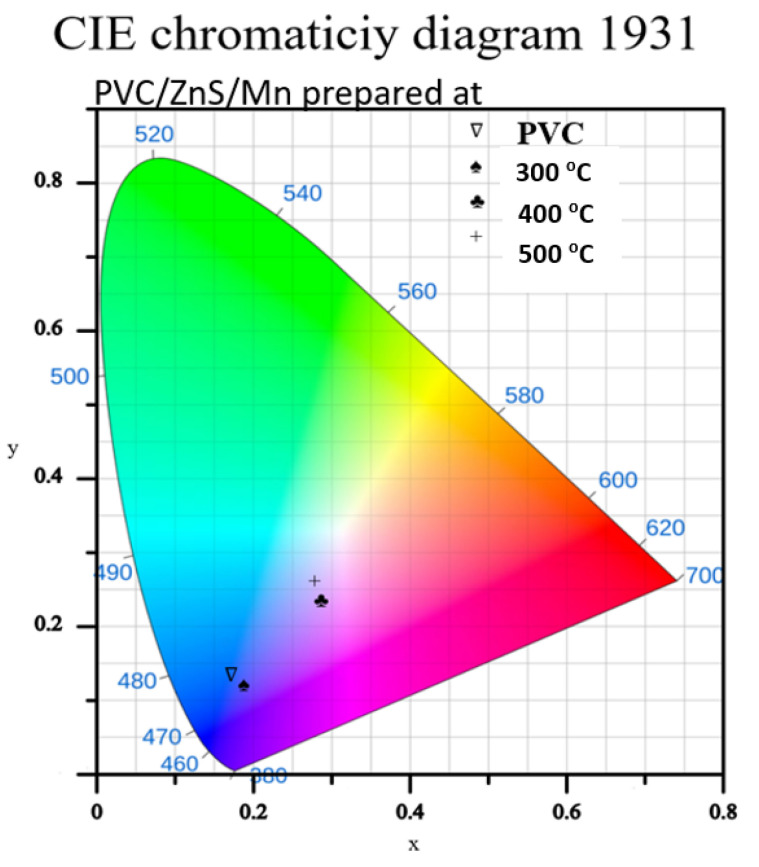
CIE diagram of pure and doped PVC polymers with ZnS/Mn prepared at different temperatures.

The Commission Internationale de l’èclairage (CIE) coordinates of the light spectrum are determined using a CIE calculator. The tristimulus values *X*, *Y*, and *Z* were first obtained, which were additionally employed to calculate the *x* and *y* chromaticity coordinates of the normalized PL emission data of the studied polymer samples. The calculated chromaticity coordinates of all the polymers for the PL data are listed in Table 3, and the corresponding CIE diagram is displayed in Figure 9.

### 3.4. Dielectric Characteristics

**Figure 10:**
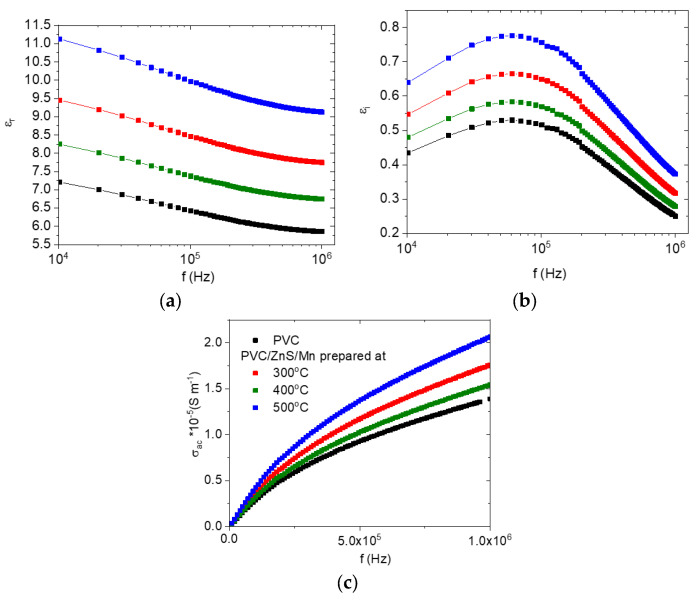
Variation in (**a**) real, (**b**) imaginary dielectric constant, and (**c**) AC conductivity for pure and doped PVC polymers with ZnS/Mn prepared at different temperatures with frequency.

**Figure 11:**
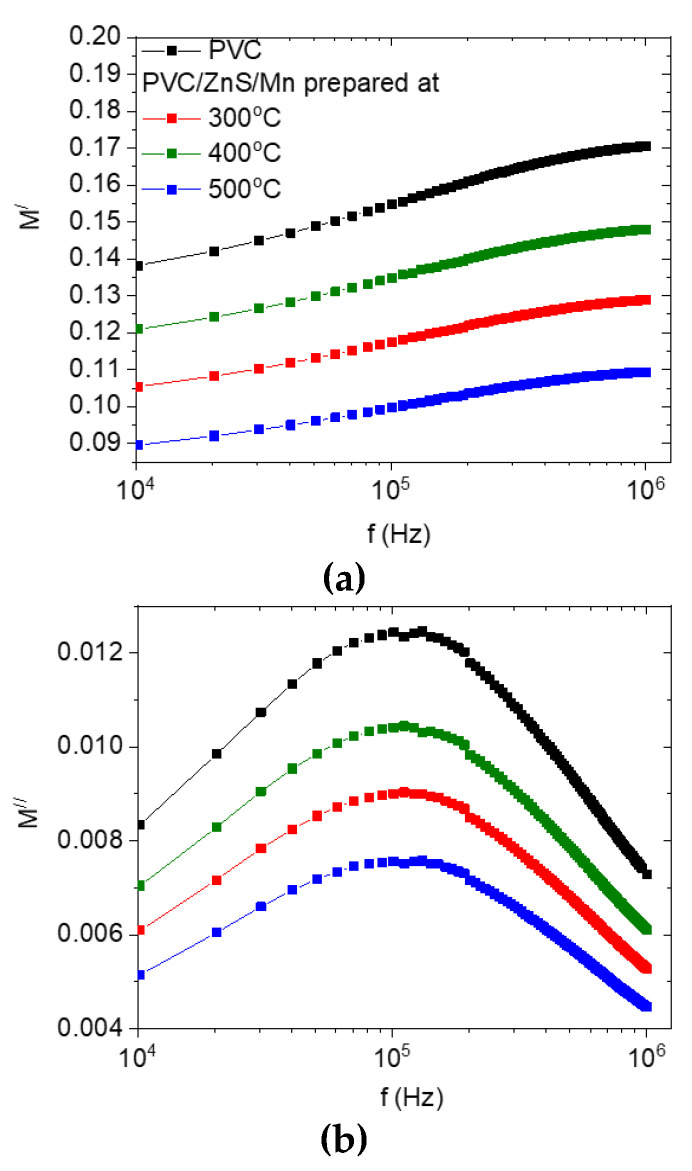
(**a**) Real and (**b**) imaginary parts of electric modulus for pure and doped PVC polymers with ZnS/Mn prepared at different temperatures.

Figure 10 depicts the frequency-dependent variation in the real (*ε′*) and imaginary (*ε″*) parts of the dielectric constant and AC conductivity of the undoped and doped PVC with ZnS/Mn prepared at different temperatures. The *ε′* for all the polymers has maximum values at lower frequencies and reduces as the frequency increases, as shown in Figure 10a. Space charge polarization is thought to be the primary reason for the large value of *ε′* at low frequencies [46]. The polarization decreased and, consequently, *ε′* [47] reduced as the frequency increased because the various dipoles inside the various polymers were incapable of following the electric field. When ZnS/Mn was added to the PVC, *ε′* increased irregularly, reaching its highest value in the nanofiller prepared at 500 °C, while it reached its lowest value in the nanofiller prepared at 400 °C. The strength of the electrostatic interaction force acting between the ZnS/Mn (formed at different temperatures) and the functional group in the PVC polymer chain that facilitates molecular movement determined the enhancement in the *ε′* values. As a result, the effective dielectric polarization of the doped polymer was increased in comparison to the undoped polymer. Moreover, the *ε″* curves for all the polymers presented the α-relaxation peak in the intermediate frequency range [48], as shown in Figure 10b. As the PVC was doped with ZnS/Mn, the values of *ε″* grew. Surface, mechanical stress, and extrinsic grain boundary effects are enhanced or weakened, respectively, in filler with smaller or larger grains [49]. As the crystalline sizes shrank, the interface area between the filler and polymer materials grew, leading to an increase in interfacial polarization and, ultimately, an increase in *ε″* [50]. The frequency-dependent variations in the AC electrical conductivity (*σ_ac_*) are shown for all samples in Figure 10c. As can be seen in the graph, as the frequency increases, so does the value of *σ_ac_*. In addition, the value of *σ_ac_* of the doped polymer enlarged owing to the addition of ZnS/Mn. The change in the *σ_ac_* value of the doped polymers is related to the modification in charge carrier concentration and mobility in the various polymers [51]. Dielectric and conductivity analysis has revealed that doped polymers can be used in the construction of electronic devices such as capacitors, organic field effect transistors, and antennas due to their performances, which are largely governed by the electrical conductivities of these hybrid dielectric materials. Figure 11 displays the real and imaginary parts of the electrical modulus (*M′*, *M″*) as a function of the frequency for all the polymers. As the frequency increased, the *M′* value increased. Due to the small contribution of electrode polarization in all the samples, *M′* is smaller at lower frequency ranges for all the doped samples [52]. The increase in the *M′* values with frequency is caused by the short-range mobility of charge carriers, which causes the conduction process. It is linked to the absence of a restoring force that directs the movement of charge carriers under the influence of an induced electric field [53]. Additionally, *M′* reduced as the ZnS/Mn-doped PVC was prepared at 500 °C, reaching its lowest value at that temperature. In addition, a relaxation peak was observed in the graph of *M″* versus frequency for all the polymers as a consequence of the conductivity process [54]. All the polymers presented the α-relaxation process. As the polymer was doped with ZnS/Mn, the place of this peak shifted. This shift is caused by variations in charge carrier movement [55].

### 3.5. DC Electrical Conductivity

**Figure 12:**
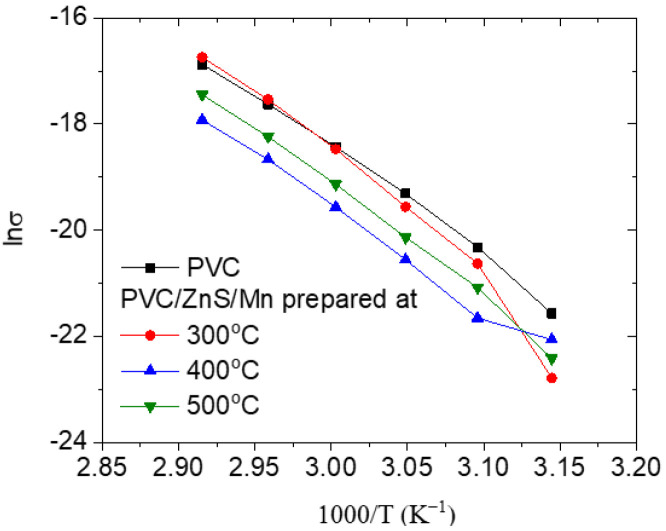
Variation in lnσ with temperature for pure and doped PVC polymers with ZnS/Mn prepared at different temperatures.

For the undoped and doped PVC prepared with ZnS/Mn at various temperatures, Figure 12 illustrates the change in ln (*σ*) with the inverted temperature (1000/T). The graph displays that, as the inverted temperature (1000/T) increases, the ln (*σ*) values for all the polymers reduce. Additionally, DC conductivity enlarged slightly when the PVC was doped with ZnS/Mn formed at 300 °C and reduced when it was doped with ZnS/Mn prepared at 400 or 500 °C. This discrepancy was determined by the ability of the nanofiller’s dispersion within the polymer matrix and the polymer–filler interaction [56].

The Arrhenius relation [56] can be used to describe the DC conductivity of a polymer as a function of the temperature:

(6) $$\sigma_{dc}=A\;\exp(-E_{A}/k_{B}T)$$

where *E_A_*, *A*, and *k_B_* are the DC conductivity activation energy, temperature independent constant (based on the polymers’ physical and chemical features), and Boltzman’s constant, respectively.

After performing the least square fitting of the DC data using the final formula, as shown in Table 2, the value of *E_A_* for each polymer can be determined. It is worth noting that the *E_A_* for the PVC (0.174 eV) was decreased to 0.165 eV as it was loaded with ZnS/Mn formed at 400 °C, while it increased to 0.187 or 0.185 eV as it was loaded with ZnS/Mn formed at 300 or 500 °C. This result may be caused by the variation in the delocalized charge carriers in different samples [57].

## 4. Conclusions

All the ZnS/Mn samples had a single phase ZnS with a zinc blende structure. FTIR analysis confirmed the occurrence of interactions between the PVC and doping substances. The PVC film lacked pores and was smooth, but it turned rough when ZnS/Mn prepared at different temperatures was added to the PVC polymer, and the degree of roughness increased with the increasing ZnS/Mn preparation temperature. The EDS data established the presence of ZnS/Mn filler over the PVC matrix. Due to the increase in absorbance upon doping, the doped polymers can be used as UV-blocking materials. The *E_dir_* and *E_in_* values increased with an increase in the nanofiller’s (ZnS/Mn) preparation temperature. When ZnS/Mn is prepared at a temperature of 300 °C, the *k* value reached its maximum value and decreased as the preparation temperature rose. The *n* value displayed the uppermost value in the PVC doped with ZnS/Mn prepared at 300 °C. When ZnS/Mn was added to PVC, *ε′* increased irregularly, reaching its highest value when the nanofiller was prepared at 500 °C, while having its lowest value when the nanofiller was prepared at 400 °C. The values of *ε″* were enlarged in the PVC doped with ZnS/Mn. The value of *σ_ac_* in the doped polymer enlarged owing to the addition of ZnS/Mn. *M′* reduced in the ZnS/Mn-doped PVC prepared at 500 °C, reaching its lowest value at that temperature. The -α-relaxation process was observed in all polymers. The DC conductivity enlarged slightly when the PVC was doped with ZnS/Mn prepared at 300 °C and reduced when it was doped with ZnS/Mn prepared at 400 or 500 °C. The activation energy of the PCV was affected by the formation temperature of the nanofiller. The doped PVC’s diverse optical and electric properties qualified them for use in a variety of optoelectronic applications.

## Figures and Tables

**Table 1:** Lattice parameter (a), crystallite size, and lattice microstrain of Zn_0.95_Mn_0.05_S (ZnS/Mn) prepared at different temperatures.

$\mathrm{ZnS}/\mathrm{Mn}\;(F\;\bar{3}4m)$			
T °C	*a* (A)	Size (nm)	Strain
300	5.3750 (7)	20	0.0115
400	5.3756	22	0.0102
500	5.3787	25	0.0075

**Table 2:** Direct and indirect optical band gaps and activation energy for PVC and PVC/ZnS/Mn polymers prepared at different temperatures.

Sample	Direct *E_g_* (eV)	Indirect *E_g_* (eV)	*E_A_* (eV)
Undoped PVC	5.41	4.52	0.174
Doped PVC with ZnS/Mn prepared at			
300 °C	4.56	3.63	0.187
400 °C	4.61	3.8	0.165
500 °C	4.67	4.08	0.185

**Table 3:** Chromaticity coordinates (x, y) for the FL spectra shown in Figure 9.

Sample	Corresponding CIE Coordinates (x, y) for Emission
Undoped PVC	(0.1707, 0.1336)
Doped PVC with ZnS/Mn prepared at	
300 °C	(0.1880, 0.1205)
400 °C	(0.2845, 0.2346)
500 °C	(0.2776, 0.2608)

## Data Availability

Data is available from the corresponding author on reasonable request.
